# Endocytic Pathways and Actin Remodeling Mediate Everolimus-Induced VE-Cadherin Disorganization and Barrier Dysfunction

**DOI:** 10.1007/s12195-025-00881-y

**Published:** 2025-12-03

**Authors:** Ken D. Brandon, Yoshi Chettri, Azkah Anjum, Kimberly M. Stroka

**Affiliations:** 1 Fischell Department of Bioengineering, University of Maryland, College Park, MD 20742 USA; 2 Marlene and Stewart Greenebaum Comprehensive Cancer Center, University of Maryland, Baltimore, MD 21201 USA; 3 Biophysics Program, University of Maryland, College Park, MD 20742 USA; 4 Center for Stem Cell Biology and Regenerative Medicine, University of Maryland, Baltimore, MD 21201 USA; 5 Fischell Department of Bioengineering, University of Maryland, 3110, A. James Clark Hall, 8278 Paint Branch Drive, College Park, MD 20742 USA

**Keywords:** VE-cadherin, Everolimus, mTOR inhibition, Endocytosis, F-actin anisotropy, Permeability

## Abstract

**Purpose:**

VE-cadherin is a key component of endothelial adherens junctions, and its disorganization contributes to vascular dysfunction. While rapamycin analogs like everolimus (EVL) are clinically linked to endothelial barrier dysfunction (EBD), the underlying molecular mechanisms remain poorly defined. This study investigates how EVL alters VE-cadherin organization, trafficking, cytoskeletal architecture, and barrier function in endothelial cells.

**Methods:**

Human umbilical vein endothelial cells (HUVECs) were treated with 500 nM EVL for 4 or 24 h. Junctional VE-cadherin organization was quantified using confocal microscopy and the Junction Analyzer Program. Cytoskeletal changes were assessed via F-actin anisotropy, and pharmacologic inhibitors (chlorpromazine, chloroquine, and brefeldin A) were used to block clathrin-mediated endocytosis, lysosomal degradation, and Golgi trafficking, respectively. Barrier function was evaluated using TEER and 4 kDa FITC-dextran transwell assays.

**Results:**

EVL reduced continuous VE-cadherin and increased punctate junctions in a time-dependent but partially reversible manner. Inhibiting endocytosis or lysosomal degradation preserved VE-cadherin continuity, while Golgi disruption blocked recovery. EVL also increased F-actin anisotropy, reflecting enhanced stress-fiber alignment within individual cells, but transiently uncoupled intracellular actin organization from coordinated cytoskeletal alignment across the monolayer. Functionally, EVL decreased TEER and increased dextran permeability by 2.24–2.63-fold, indicating significant barrier disruption.

**Conclusions:**

EVL compromises endothelial barrier integrity by promoting VE-cadherin internalization and lysosomal degradation, accompanied by cytoskeletal remodeling and a Golgi-dependent, partial restoration of junctional VE-cadherin. These findings highlight endocytic, degradative, and Golgi-mediated trafficking pathways as key modulators of EVL-induced endothelial barrier dysfunction and provide mechanistic insight into the vascular effects of rapalog-based mTOR inhibition.

**Supplementary Information:**

The online version contains supplementary material available at 10.1007/s12195-025-00881-y.

## Introduction

Endothelial cells are central to vascular homeostasis and barrier function, maintaining selective permeability, supporting immune surveillance, regulating vascular tone, and responding to biochemical and biomechanical cues through dynamic cell–cell junctions [1–5]. A critical determinant of this barrier function is vascular endothelial (VE)-cadherin, an adherens junction protein that anchors endothelial cell–cell contacts. Disruption of VE-cadherin organization compromises junctional integrity and can lead to endothelial barrier dysfunction (EBD), a pathological increase in vascular permeability observed in diseases such as atherosclerosis, cancer, infections, and trauma [6].

In addition to disease-associated mechanisms, therapeutic agents can also trigger EBD. Everolimus (EVL), a rapamycin analog that inhibits the mechanistic target of rapamycin (mTOR), is widely used in transplant medicine [7, 8] and drug-eluting stents (DES) [9]. However, EVL has been associated with adverse vascular effects. In an in vivo model of EVL-eluting stents, reduced VE-cadherin coverage at endothelial cell–cell junctions, increased monocyte adhesion at VE-cadherin–negative junctions, and delayed endothelial healing have been reported, underscoring the need to define the molecular basis of EVL-induced EBD [10]. Beyond stent-related pathology, clinical trials, observational studies, and drug safety databases consistently report that EVL use in transplant recipients is associated with edema, most commonly presenting as lower limb swelling [11–14]. While the clinical effects of EVL on vascular integrity are increasingly recognized, the molecular mechanisms by which it disrupts endothelial junctions remain incompletely defined.

Previous in vitro studies have linked EVL and other rapalogs to VE-cadherin disruption [15–17], including its dissociation from junctional partners such as p120-catenin [18], representing an essential step toward understanding the contribution of rapalog-based mTOR inhibition to endothelial barrier impairment. Subclassification of VE-cadherin morphology into continuous, punctate, and perpendicular conformations has been shown to correlate with monolayer maturity and barrier function, supporting the value of this approach in identifying subtle yet functionally meaningful junctional disruptions [19–21]. Quantitative morphological analysis can therefore enhance resolution beyond binary assessments of protein presence or absence and offer insight into dynamic remodeling events that govern endothelial integrity.

While VE-cadherin internalization has been observed following rapamycin analog treatment, direct experimental evidence linking this process to specific endocytic pathways—and defining the downstream morphological consequences—remains limited. In parallel, actin remodeling is a critical regulator of endothelial cell shape and junctional stability. Although EVL-induced stress fiber formation has been reported [17], the impact of EVL on actin alignment—an important determinant of cytoskeletal tension—has not been quantitatively assessed. Since actin architecture contributes to both the maintenance and remodeling of adherens junctions, evaluating these changes may offer additional insight into the cytoskeletal mechanisms underlying barrier disruption.

This study focuses on whether clathrin-mediated endocytosis (CME) is a key pathway contributing to VE-cadherin junctional disruption and employs a morphometric framework to characterize junctional reorganization in response to EVL. To better define the underlying features of EVL-induced junctional disruption, we used a quantitative approach to examine temporal changes in VE-cadherin organization, trafficking, cytoskeletal remodeling, and barrier function. We hypothesized that EVL impairs endothelial barrier integrity by promoting VE-cadherin internalization via endocytosis and subsequent lysosomal degradation, accompanied by actin reorganization that disrupts cell–cell junctions. To test this, we primarily treated human umbilical vein endothelial cells (HUVECs) with EVL and quantified changes in VE-cadherin conformations, cell morphology, and F-actin organization. In addition, we included human brain microvascular endothelial cells (HBMECs) in supplemental experiments to examine heterogeneity in endothelial cell responses. In HUVECs specifically, pharmacologic inhibition of trafficking pathways using brefeldin A (BFA), chlorpromazine, and chloroquine was employed to dissect underlying mechanisms. Finally, we assessed functional consequences using TEER and transwell permeability assays in EVL-treated HUVEC monolayers.

## Materials and Methods

### Cell Culture

To model endothelial barrier dynamics in vitro, we used primary human endothelial cells from distinct vascular beds. Human umbilical vein endothelial cells (HUVECs) and human brain microvascular endothelial cells (HBMECs) were cultured under the conditions described below. Please see the Major Resources Table in the Supplemental Materials for additional details on key reagents, cell lines, and instruments.

HUVECs were cultured in Endothelial Cell Growth Medium MV2, which includes fetal calf serum (0.05 mL/mL), recombinant human epidermal growth factor (5 ng/mL), basic fibroblast growth factor (10 ng/mL), insulin-like growth factor (Long R3 IGF, 20 ng/mL), vascular endothelial growth factor 165 (0.5 ng/mL), ascorbic acid (1 µg/mL), and hydrocortisone (0.2 µg/mL), as part of the MV2 Supplement Mix. Penicillin/streptomycin (1%) was added separately. HUVECs were confirmed mycoplasma-free upon arrival, cryopreserved at passage 3, and used between passages 4–6.

HBMECs were cultured on 0.1% gelatin-coated flasks in RPMI-1640 medium supplemented with 20% fetal bovine serum, 1% penicillin/streptomycin, 2 mM L-glutamine, 30 µg/mL endothelial cell growth supplement, and 100 µg/mL heparin, as previously described [22]. HBMECs were confirmed mycoplasma-free upon arrival and used at passages 7–10. All cells were maintained at 37 °C in a humidified incubator with 5% CO_2_.

### Substrate Coating and Experimental Conditions

All experimental substrates were pre-coated with fibronectin to promote cell adhesion and consistent monolayer formation. On Day 0, glass-bottom 24-well plates or 12-well transwell inserts were coated with fibronectin (100 µg/mL for 24-well plates; 10 µg/mL for transwells) in DPBS (Ca^2^⁺/Mg^2^⁺), incubated at room temperature for 1 h, rinsed with PBS, and equilibrated with pre-warmed complete medium. Endothelial cells were seeded at 9.5 × 10^4^ cells/well (24-well) or 1.5 × 10^5^ cells/insert (transwells) and cultured for 48 h to establish confluence. EVL was applied at 500 nM for either 4 or 24 h, consistent with concentrations used in previous in vitro studies [16, 18, 23]. Prior work demonstrates that this dose effectively inhibits mTORC1, as evidenced by decreased p-p70S6K, and partially inhibits mTORC2, as reflected by reduced p-Akt levels in endothelial cells [16].

In select experiments, chlorpromazine (25 µM), chloroquine (100 µM), and BFA (3.6 µM) were used to inhibit CME, lysosomal degradation of internalized VE-cadherin, and Golgi-dependent trafficking, respectively. The concentration of chlorpromazine was adopted from prior studies in endothelial systems, which demonstrated that 25 µM reduced membrane particle uptake in HUVECs by approximately 95% compared to control [24]. Chloroquine at 100 µM was selected based on its previously reported efficacy in blocking lysosomal degradation of internalized VE-cadherin [25]. Given the limited specificity of these agents, all findings were interpreted with consideration of their broader cellular effects. The concentration of BFA was determined through an in-house dose-response experiment assessing Golgi disruption using GM130 and Golgin-97 as markers (data not shown), with 3.6 µM effectively disrupting Golgi organization in HUVECs. Overnight treatment with BFA resulted in cytotoxic effects; therefore, HUVECs were only exposed to acute (4 h) incubation periods, well in advance of any detectable cytotoxicity.

Endothelial cells were pre-treated with chlorpromazine and chloroquine for 30 min prior to EVL addition. For delayed co-treatment, BFA was added 4 h after EVL. Control conditions received matched vehicle treatments (DMSO or sterile water). For immunofluorescence experiments, all samples were fixed at the same endpoint (24 h after the first EVL addition). To achieve this, EVL was added to the 24 h treatment group at the start of the experiment and to the 4 h group 20 h later, such that both groups were fixed simultaneously after their respective treatment durations.

### Immunofluorescence Staining

To visualize protein localization and junctional conformations, cells were fixed with 2% paraformaldehyde for 20 min at room temperature and washed with PBS. Permeabilization was performed using 1% Triton X-100 for 5 min (or 0.25% Triton X-100 for CME assays). Blocking was carried out with 2% BSA for 1 h at room temperature. Cells were incubated with primary antibodies against VE-cadherin (1:100) and GM130 (1:3200) overnight at 4 °C. Phalloidin–Alexa Fluor 568 (0.1 µM) was applied for 30 min at room temperature. Following primary incubation, cells were incubated with appropriate secondary antibodies (1:1000) for 1 h at room temperature. Nuclei were counterstained with Hoechst 33342 (2 µg/mL) for 5 min. Plates were stored in PBS at 4 °C until imaging.

### Microscopy

All imaging was performed to capture high-resolution spatial data on junctional and cytoskeletal organization. Confocal imaging was conducted using an Olympus FV3000 microscope equipped with a 60 × oil immersion objective (NA 1.4). Z-stack images were acquired at 0.2 µm intervals with a 1024 × 1024 pixel resolution. Imaging parameters were optimized during each session using the microscope’s range indicator mode, which highlights saturated pixels in red on a grayscale background, to ensure that signal was captured within the linear dynamic range. Consistent procedures were followed to ensure signal linearity and comparability across conditions.

Z-stack images were post-processed using maximum intensity projection along the z-axis in the Olympus FV3000 acquisition software to generate 2D representations of VE-cadherin localization. All images were subsequently processed using the same thresholding workflow (Fig. S1). Representative images were adjusted equally for brightness and contrast across all conditions for display purposes only. All quantitative analyses were performed on raw, unprocessed images unless otherwise stated.

### Junction and Morphology Quantification

Quantitative analysis of VE-cadherin junction organization was performed using the Junction Analyzer Program (JAnaP), a semi-automated image analysis tool developed in-house and described previously [19]. The software is publicly available at https://github.com/StrokaLab/JAnaP. Briefly, perimeters of randomly selected cells were delineated using manually placed “waypoints,” which generate a cell-edge skeleton based on VE-cadherin–labeled pixels at cell–cell contact sites. Cells along image borders or those partially out of frame were excluded. Per image, approximately 10–20 cells were analyzed, with 4–6 images evaluated per condition for each of ≥ 3 independent biological replicates. Intensity thresholds were established a priori in the JAnaP Jupyter Notebook environment to minimize background noise and were maintained consistently across all datasets (red channel = 20; green channel = 35) (see Fig. S1).

JAnaP computes multiple cell morphology parameters, including area, perimeter, circularity, and solidity, and classifies VE-cadherin junctions into continuous, perpendicular, or punctate conformations. Continuous junctions were defined as linear segments extending along the cell edge for > 15 pixels (~1.3 µm in our imaging configuration), perpendicular junctions were defined as non-continuous junctions exhibiting an aspect ratio > 1.2, and remaining junctional fragments were categorized as punctate. Cell morphology parameters were also quantified using the JAnaP. Circularity was calculated as 4π × (area/perimeter^2^), where a value of 1 represents a perfect circle and decreasing values correspond to greater cell elongation or irregularity. Solidity was calculated as (cell area)/(convex hull area), where a value of 1 indicates a fully convex cell perimeter, and lower values indicate increased boundary irregularity resulting from protrusions or indentations.

Output data were aggregated to determine the proportional representation of each junction type per cell and were used to compute summary statistics across ≥ 3 independent experiments. Total VE-cadherin percent coverage was defined as the sum of all identified junctional conformations along the cell perimeter, such that 100% represented the entire cell perimeter. Values were expressed as the mean proportion of continuous, punctate, and perpendicular conformations across the sample cell population. All images used for analysis were acquired using consistent confocal settings and post-processed using a uniform workflow to ensure fluorescence intensity was comparable across experimental conditions. This approach enabled objective assessment of VE-cadherin localization and conformation in response to treatment.

### F-actin Anisotropy and Orientation Analysis

To assess cytoskeletal organization, F-actin alignment was quantified using the FibrilTool plugin in Fiji/ImageJ, as previously described [26]. Individual endothelial cells were manually outlined using VE-cadherin staining as a reference. Brightness and contrast were uniformly adjusted across all conditions, as recommended by the developers of FibrilTool, to enhance fibril visibility without pixel saturation.

For each field of view (FOV), FibrilTool generated per-cell anisotropy coefficients (A) and mean fibril orientation angles (− 90° to 90°, relative to the x-axis). The anisotropy coefficient (A) ranges from 0 (random orientation) to 1 (perfect alignment). The range of anisotropy values observed in this study (0.1–0.5) is consistent with previously reported values for biological fibrillar structures analyzed using FibrilTool [26–28].

All FOVs were randomly sampled from confluent monolayers and were not spatially adjacent. To evaluate coordinated alignment within each FOV, alignment strength (R) was calculated from F-actin orientation data obtained using FibrilTool. Per-cell orientation angles were converted from degrees to radians, doubled to account for axial symmetry, and used to compute the cosine and sine components. The mean cosine and sine values were then averaged across all cells within each FOV, and alignment strength (R) was calculated as:

$$R = \sqrt{({\overline{\text{cos }2\theta )}}^{2}+({\overline{\text{sin }2\theta )}}^{2}}$$

where R = 1 indicates that all cells within the FOV share a common orientation and R = 0 indicates random or opposing orientations. A Rayleigh test was also applied to determine whether orientations were significantly clustered around a preferred axis. This analysis allowed the distinction between local stress-fiber alignment (anisotropy) and a more global orientation coherence (R) across the imaged field [29].

### Barrier Function Assays

To evaluate monolayer barrier function, HUVECs were seeded on 12-well transwell inserts (0.4 µm pores, 150,000 cells/insert) and cultured for 48 h. Transendothelial electrical resistance (TEER) was measured using an EVOM3 voltohmmeter with STX4-Plus electrodes (World Precision Instruments), normalized to blank (cell-free) inserts, and reported in Ω·cm^2^. Because TEER values in HUVEC monolayers are low and susceptible to measurement variability, we minimized noise by equilibrating plates to room temperature for at least 10 min, preventing bubble formation, rinsing electrodes between wells, standardizing insertion depth and orientation, and gently positioning the STX4-Plus probe. Two consecutive readings were collected per insert and averaged.

To account for potential measurement bias, TEER was interpreted alongside a complementary permeability assay using FITC–dextran, which provided a rate-based readout less sensitive to probe handling. For permeability assays, FITC-dextran (4 kDa; 20 µg/mL) was added to the apical chamber. Basolateral samples (150 µL) were collected every 15 min for 1 h, and fluorescence was measured using a Tecan Spark plate reader (Ex 485 nm/Em 535 nm). After each collection, 150 µL of fresh media was added to maintain constant volume. Clearance volume (µL) at each time point was calculated as previously described [30]:

$$Clearance Volume = \frac{(Basolateral volume \times Basolateral fluorescence intensity)}{(Apical fluorescence intensity)}$$

The clearance rate (Cr) was defined as the slope of the clearance volume (CV) versus time curve, obtained by linear regression in GraphPad Prism 10 for both cell-containing inserts (${m}_{c}$) and blank controls (${m}_{i}$). When clearance volumes were normalized to the blank, the resulting slope represented the clearance rate constant (min⁻^1^). Inverse permeability was calculated using the following equation:

$$\frac{1}{Pe}=\frac{1}{{m}_{c}}-\frac{1}{{m}_{i}}$$

The final permeability coefficient (Pe) was normalized to the surface area of the transwell membrane and reported in units of cm/min.

### Statistical Analysis

All statistical analyses and figure generation were performed using GraphPad Prism 10. Data normality was assessed using the D’Agostino–Pearson test. For normally distributed data, one-way ANOVA with Tukey’s multiple comparisons test was applied; for non-normal data, the Kruskal–Wallis test with Dunn’s post hoc correction was used. The Friedman test with Dunn’s post hoc correction was applied for non-parametric repeated-measures analyses across experiments (e.g., Fig. S3B). Linear regression was used to compare clearance slopes. Spearman’s rank correlation coefficient (r) was computed to evaluate the relationship between per-FOV median anisotropy and alignment strength (R) within each treatment condition. Outliers were identified using the ROUT method (Q = 1%); outliers were detected only in the control group (Fig. 6C), and predefined removal criteria were applied uniformly across all groups.

**Fig. 1:**
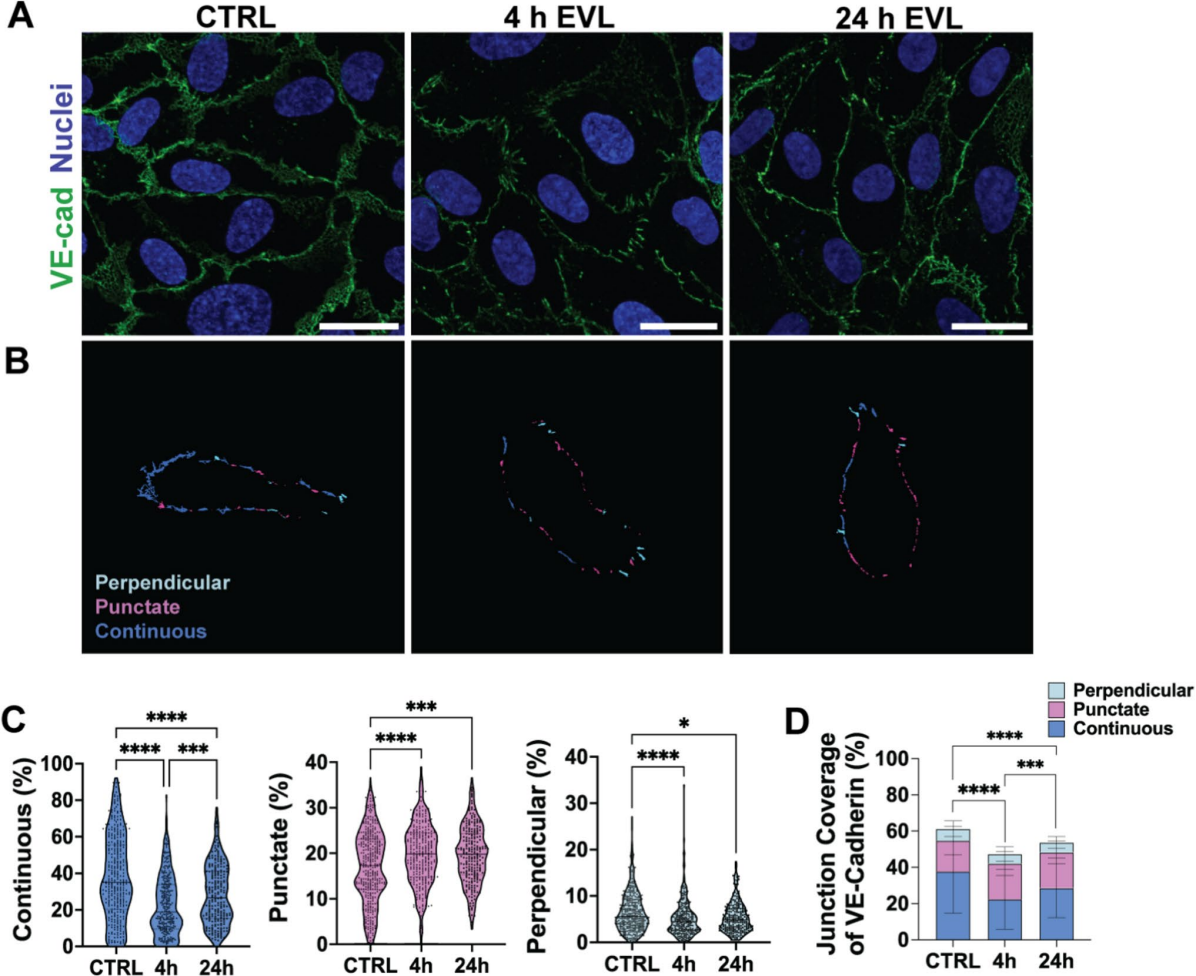
Everolimus alters VE-cadherin junction organization in HUVECs. **A**, **B** Representative images of HUVECs stained for VE-cadherin after 4 h and 24 h of everolimus (EVL) treatment compared to control. **C** Quantification of VE-cadherin conformations—continuous, punctate, and perpendicular—using the Junction Analyzer Program (JAnaP) (n = 237–357 cells per condition, three independent experiments). **D** Population-averaged junction coverage of VE-cadherin, shown as the mean proportions of continuous, punctate, and perpendicular conformations across the sample cell population. Data represent mean ± SD from three independent experiments. Statistical analysis was performed using a Kruskal–Wallis test with Dunn’s post hoc correction. Scale bars: 25 µm. Images acquired at 60 × and cropped; calibration preserved. *p < 0.05; **p < 0.01; ***p < 0.001; ***p < 0.0001.

**Fig. 2:**
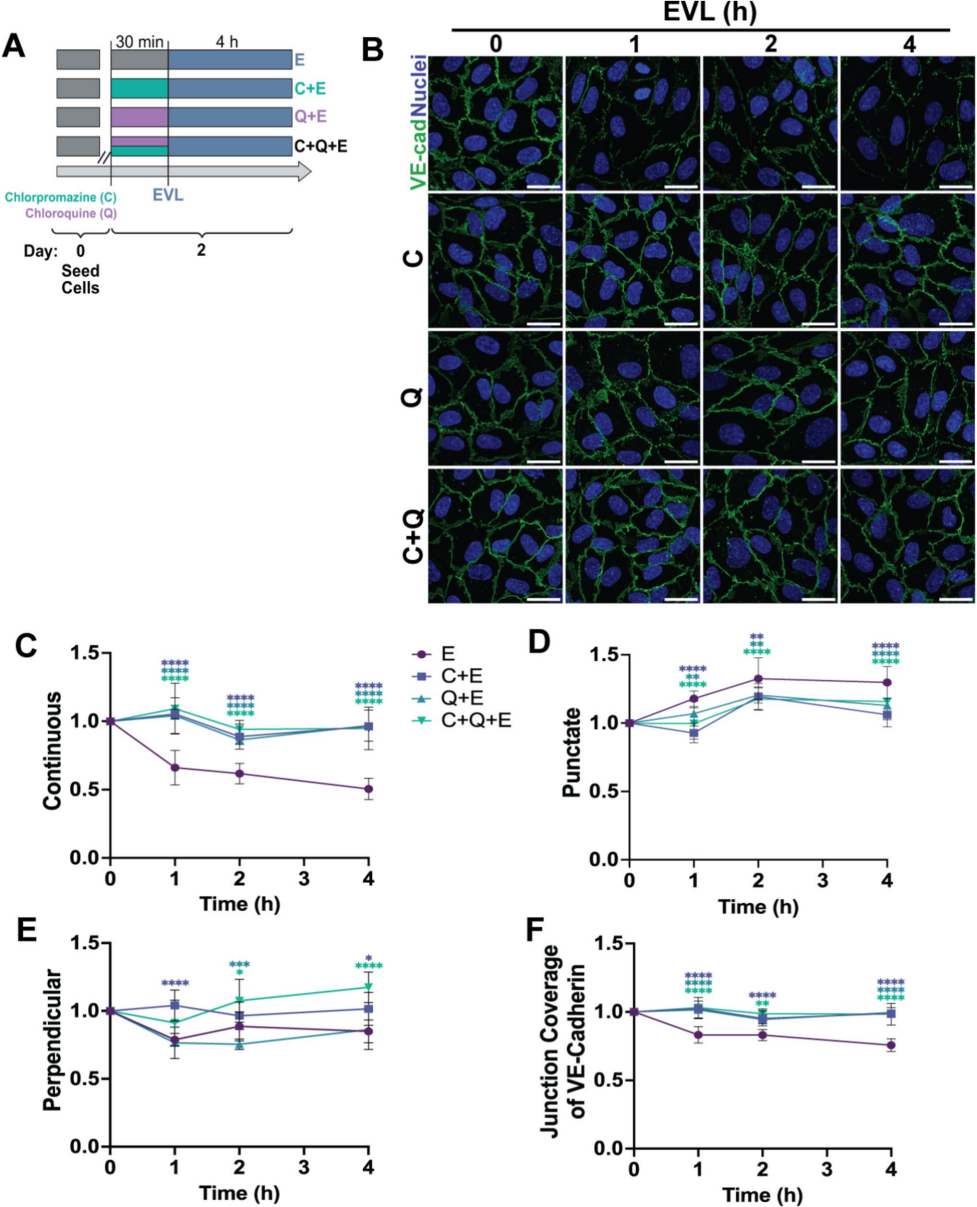
Inhibition of clathrin-mediated endocytosis (CME) or lysosomal degradation prevents EVL-induced VE-cadherin disruption. **A** Experimental design: HUVECs were pretreated with chlorpromazine (C), chloroquine (Q), or both, followed by EVL exposure for 0–4 h. **B** Representative VE-cadherin images. **C**–**F** Quantification of junction conformations normalized to time 0: continuous (**C**), punctate (**D**), perpendicular (**E**), and population-averaged VE-cadherin junction coverage (**F**). Color-coded treatment groups in (**C**) apply across all panels. Plots represent pooled single-cell measurements from three independent experiments (n = 330–405 cells per condition; approximately 50–160 cells per experiment). Data represent mean ± SEM from three independent experiments. Statistical analysis was performed using a Kruskal–Wallis test with Dunn’s post hoc correction. Statistics shown compare EVL-only (**E**) to each inhibitor group; full pairwise comparisons are provided in Table S2. Scale bars: 25 µm. Images acquired at 60× and cropped; calibration preserved. *p < 0.05; **p < 0.01; ***p < 0.001; ****p < 0.0001

Quantification for single-cell analyses (Figs. 1, S2, 3, 4, and S3A) was performed at the individual cell level, and data from ≥ 3 independent experiments per condition were pooled to preserve biologically relevant cell-to-cell heterogeneity inherent to primary pooled HUVECs (ATCC; derived from ~ 10 donors). For field-of-view– or experiment-level analyses (Figs. 2, 5, 6, and S3B), summary values were computed per FOV or per experiment prior to statistical testing. Because pooling can increase the apparent sample size, stringent thresholds were applied when drawing conclusions (***p < 0.001, ****p < 0.0001), although all statistical comparisons are reported for transparency (*p < 0.05; **p < 0.01; ***p < 0.001; ****p < 0.0001). Data are presented as mean ± SEM unless otherwise noted.

## Results

### Everolimus Alters VE-Cadherin Junction Organization in Human Umbilical Vein Endothelial Cells

Proper VE-cadherin formation and stability are essential for maintaining endothelial barrier integrity. Although prior studies have reported that rapamycin analogs reduce VE-cadherin expression, they have primarily provided qualitative descriptions of junction morphology based on low-resolution fluorescence imaging, which limits structural interpretation [15, 18]. To address this gap, we used high-resolution confocal microscopy and quantitative image analysis to examine how EVL affects VE-cadherin organization.

HUVECs seeded on fibronectin-coated glass were treated with EVL for 4 or 24 h and immunostained for VE-cadherin (Fig. 1A, B). Quantification using JAnaP revealed a significant reduction in the continuous VE-cadherin conformation, from 37.8 ± 1.2% in controls to 22.3 ± 1.0% at 4 h and 28.6 ± 1.1% at 24 h (Fig. 1C). This disruption was accompanied by an increase in the punctate conformation and a modest decrease in the perpendicular conformation. Population-averaged VE-cadherin junction coverage declined, from 61.3 ± 1.1% in controls to 47.3 ± 1.0% at 4 h and 53.7 ± 0.9% at 24 h (Fig. 1D).

A similar pattern was observed in HBMECs, with EVL reducing the continuous VE-cadherin conformation, increasing the punctate conformation, and slightly decreasing the perpendicular conformation (Fig. S2). However, unlike HUVECs—which exhibited a pronounced disruption at 4 h with partial recovery by 24 h (p = 0.0001)—the effects in HBMECs were more sustained, with no significant difference between the two time points. Together, these findings demonstrate that EVL disrupts VE-cadherin continuity in a time-dependent but partially reversible manner in HUVECs, with more persistent effects in HBMECs, suggesting cell type-specific responses.

### Pharmacologic Inhibition of Endocytic and Lysosomal Pathways Attenuates Everolimus-Induced VE-Cadherin Disruption

Having observed that EVL disrupts VE-cadherin junction organization, we next asked whether this reduction in junctional VE-cadherin resulted from endocytic internalization. Given that VE-cadherin internalization can occur through CME following p120-catenin dissociation [25, 31], and may also undergo downstream lysosomal degradation, including through autophagy-associated pathways [32], we examined the contribution of both processes. HUVECs were pretreated with chlorpromazine, chloroquine, or both, prior to EVL exposure (Fig. 2A). HUVECs were then treated with EVL for 0, 1, 2, or 4 h and immunostained for VE-cadherin (Fig. 2B). Treatment with chlorpromazine or chloroquine attenuated the loss of continuous VE-cadherin conformation induced by EVL exposure (Fig. 2C). The increase in punctate VE-cadherin conformations observed with EVL alone was less pronounced in cells pretreated with either agent (Fig. 2D), while perpendicular conformations showed no consistent pattern across treatment groups or timepoints (Fig. 2E). Total VE-cadherin coverage, which declined notably at the 4 h time point under EVL treatment, was preserved with chlorpromazine and/or chloroquine (Fig. 2F; see Table S1 for full statistical comparisons). These findings suggest that pharmacologic disruption of endocytic or lysosomal pathways is sufficient to attenuate EVL-induced VE-cadherin internalization and junctional disruption.

### Golgi Trafficking Supports VE-Cadherin Continuity and Partially Counteracts Everolimus-Induced Junctional Disruption

**Fig. 3:**
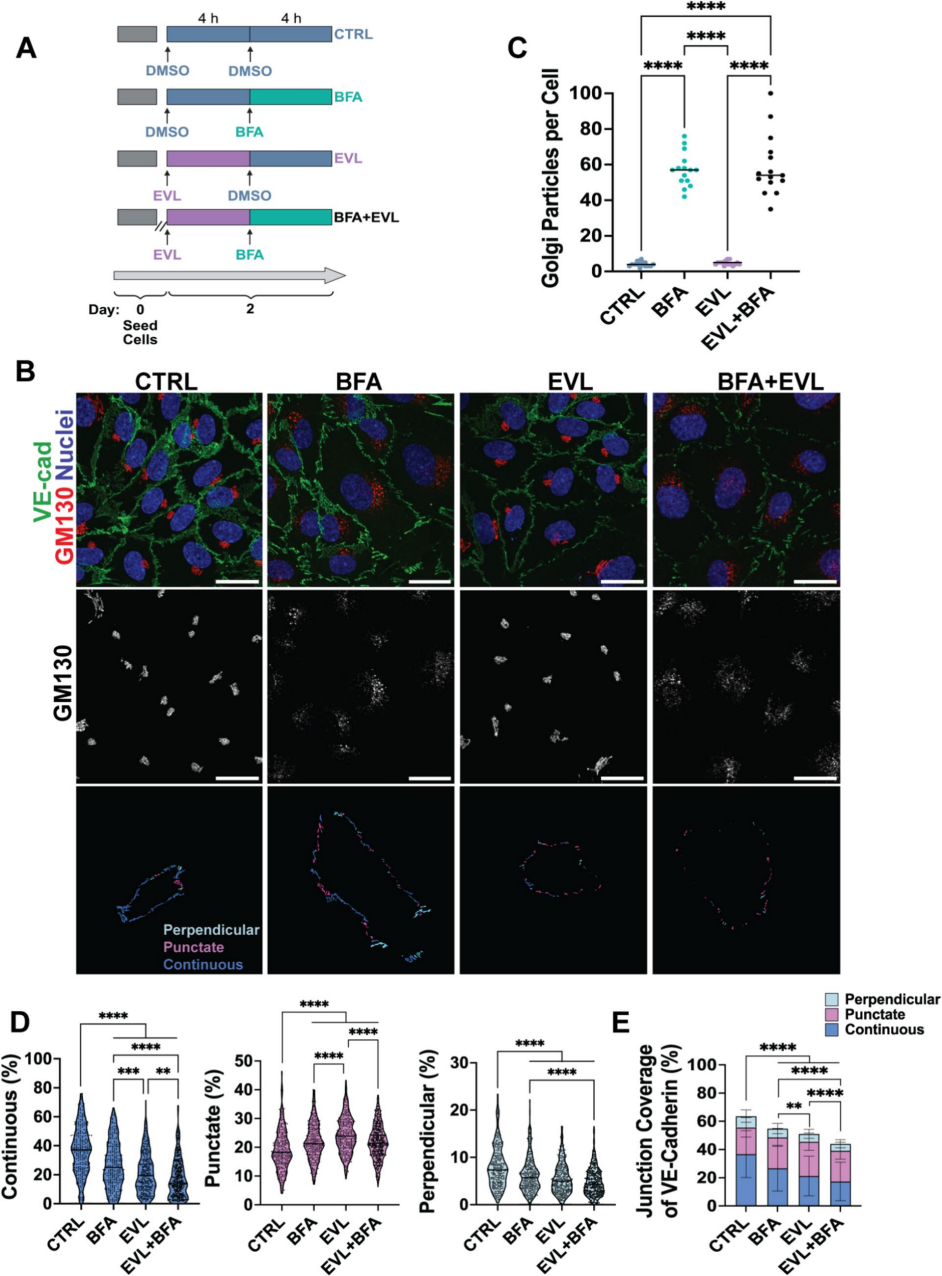
Golgi trafficking supports VE-cadherin continuity and counters EVL-induced junctional disruption. **A** Experimental design: HUVECs were pretreated with vehicle or EVL for 4 h, followed by vehicle or brefeldin A (BFA; Golgi trafficking inhibitor) for an additional 4 h. **B** Representative images of HUVECs stained for VE-cadherin (green), GM130 (red), and nuclei (blue). Middle row: GM130 alone. Bottom row: color-coded VE-cadherin conformations. **C** Quantification of GM130-positive Golgi puncta per cell (five images per condition per experiment; three independent experiments). **D**–**E** Quantification of VE-cadherin junction conformations and population-averaged junction coverage using JAnaP (n = 286–306 cells pooled across three independent experiments). Data represent mean ± SD from three independent experiments. Statistical analysis was performed using a Kruskal–Wallis test with Dunn’s post hoc correction. Scale bars: 25 µm. Images acquired at 60× and cropped; calibration preserved. *p < 0.05; **p < 0.01; ***p < 0.001; ****p < 0.0001

The partial recovery of VE-cadherin continuity within 24 h may reflect not only reduced endocytosis over time but also membrane-directed trafficking of VE-cadherin from intracellular pools [33]. To assess whether Golgi-mediated trafficking contributes to this early recovery phase, HUVECs were treated with BFA, a disruptor of ER-to-Golgi transport, for 4 h following an initial 4 h EVL exposure—the timepoint we identified as the peak of VE-cadherin disruption (Fig. 3A). Additional groups were treated with BFA alone or EVL alone to distinguish their individual and combined effects. Cells were immunostained for VE-cadherin and the Golgi marker GM130 (Fig. 3B). We quantified the number of GM130-positive Golgi puncta per cell, with BFA-treated groups showing a significant increase in puncta, indicative of the expected disruption of Golgi organization (Fig. 3C). VE-cadherin junctional organization and population-averaged junction coverage were also quantified (Fig. 3D–E).

As expected, BFA alone significantly reduced VE-cadherin continuity and total coverage, supporting a role for Golgi trafficking in VE-cadherin maintenance. EVL alone (8 h total treatment) caused a more substantial reduction in continuous VE-cadherin and a greater increase in punctate junctions compared to both control and BFA-treated groups. Co-treatment with EVL and BFA led to the most pronounced loss of continuous VE-cadherin and total coverage, indicating that BFA prevented the partial recovery observed after peak disruption. These findings suggest that Golgi-mediated trafficking contributes to VE-cadherin restoration in the early phase following EVL-induced junctional disruption.

### Everolimus Alters Endothelial Cell Morphology in a Time-Dependent Manner

**Fig. 4:**
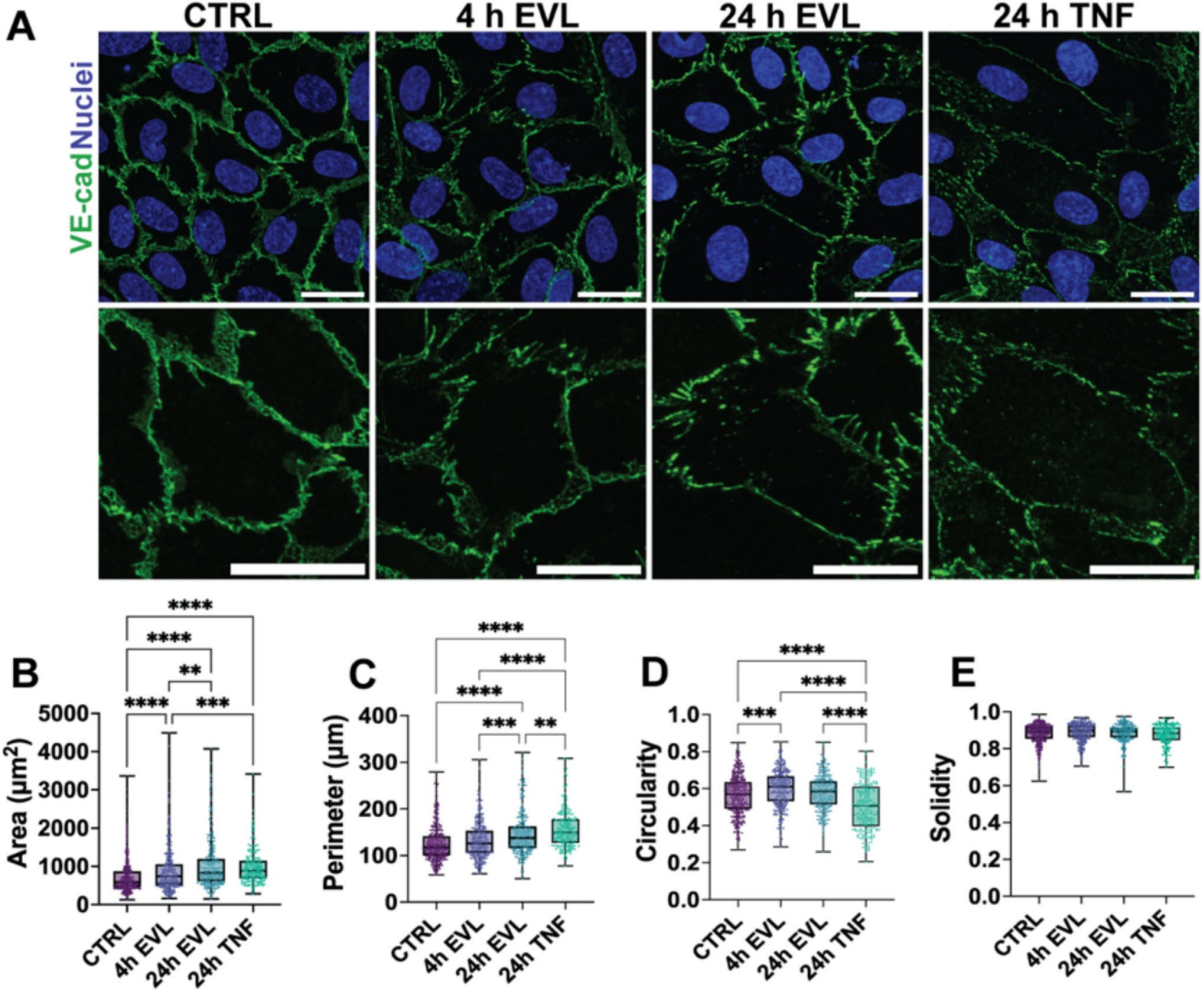
EVL induces time-dependent morphological changes in endothelial cells. **A** Representative images of HUVECs stained for VE-cadherin (green) and nuclei (blue) following CTRL, EVL (4 h, 24 h), or TNF-α (24 h) treatment. Insets: zoomed view of cell–cell junctions. **B**–**E** Quantification of cell area (**B**), perimeter (**C**), circularity (**D**), and solidity (**E**). Box-and-whisker plots represent pooled single-cell measurements from three independent experiments (n = 192–358 cells per condition). Data represent mean ± SD. Statistical analysis was performed using a Kruskal–Wallis test with Dunn’s post hoc correction. Scale bars: 25 µm. Images acquired at 60X and cropped; calibration preserved. *p < 0.05; **p < 0.01; ***p < 0.001; ****p < 0.0001

While we established that EVL disrupts VE-cadherin continuity and that Golgi-mediated trafficking contributes to its partial restoration, we also observed notable changes in overall cell morphology. Given that endothelial barrier function depends on both cell–cell junctions and the organization of cell shape and cytoskeletal architecture [34, 35], this raised the possibility that EVL’s effects extend beyond junctional disassembly to broader mechanical remodeling. To test this, we quantitatively assessed whether EVL-induced junctional disruption is accompanied by changes in HUVEC morphology (Fig. 4A). Because this phenomenon has already been demonstrated in HUVECs in response to physiological concentrations of TNF-α (25 ng/mL)—specifically, an increase in cell area and a more elongated morphology [36]—TNF-α was used as a positive control to gauge barrier-compromising morphological changes.

EVL treatment led to a progressive increase in both cell area and perimeter over time, with changes at 24 h comparable to those induced by our TNF-α positive control (Fig. 4B, C). A transient increase in cell circularity was noted at 4 h but returned to baseline levels by 24 h, whereas cell solidity—a metric of how smooth versus irregular a cell boundary is—remained unchanged across treatment conditions (Fig. 4D, E). These findings demonstrate that EVL induces time-dependent morphological changes in endothelial cells, including increased spreading and transient alterations in cell circularity.

### Everolimus Increases F-actin Anisotropy Without Promoting Coordinated Alignment Across the Endothelial Monolayer

**Fig. 5.**
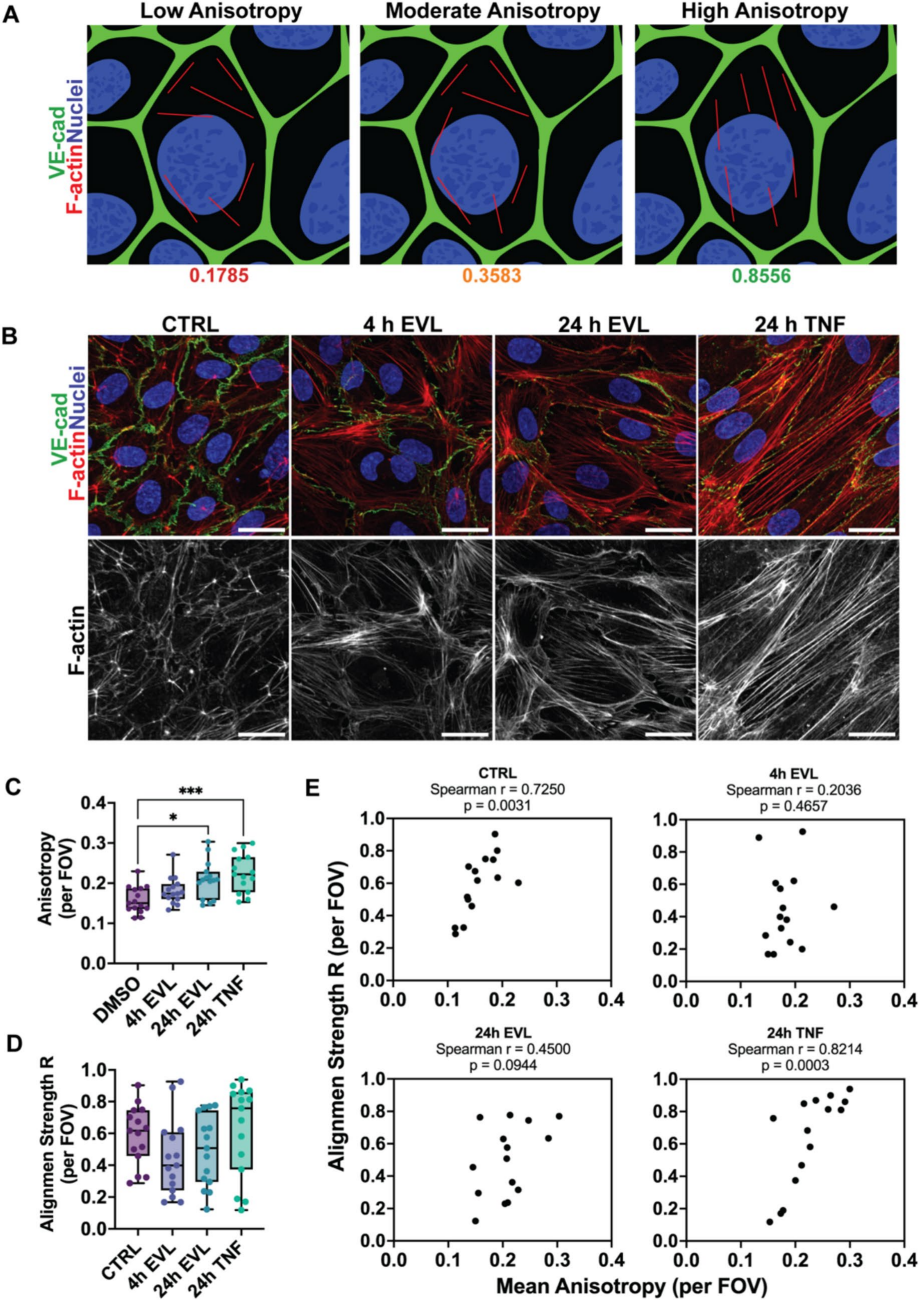
Everolimus increases F-actin anisotropy without promoting coordinated alignment across the endothelial monolayer. **A** Schematic illustrating representative anisotropy values with corresponding orientation vectors derived from FibrilTool analysis. **B** Representative confocal images of HUVECs stained for VE-cadherin (green), F-actin (red), and nuclei (blue) following control (CTRL), everolimus (EVL; 4 h or 24 h), or TNF-α (24 h) treatment. Corresponding grayscale panels display F-actin alone. **C** Quantification of mean F-actin anisotropy per field of view (FOV) (n = 15 FOVs per condition, pooled from 3 independent experiments). **D** Alignment strength (R) per FOV, calculated from the circular distribution of F-actin orientation angles within each field, represents the degree of directional coherence among neighboring cells. **E** Correlation between per-FOV anisotropy and alignment strength (R) for each treatment condition. Spearman’s rank correlation coefficient (r) and p-values are indicated. Scale bars: 25 μm. Data represent mean ± SD; statistical significance determined by Kruskal–Wallis test with Dunn’s post hoc correction. *p < 0.05, **p < 0.01, ***p < 0.001, ****p < 0.0001.

Given that EVL has been shown to promote actomyosin-driven stress fiber formation in endothelial cells [17], and that we observed EVL-induced morphological changes marked by increased cell spreading, we sought to further characterize the underlying cytoskeletal remodeling. F-actin anisotropy reflects how coherently actin filaments are organized within individual cells, serving as a quantitative measure of intracellular fiber bundling. Relatively higher anisotropy values reflect stronger stress-fiber alignment, whereas lower values indicate a more disordered cortical actin network (Fig. 5A).

F-actin anisotropy (local fiber order) increased in a time-dependent manner following EVL treatment, with 24 h EVL reaching levels comparable to TNF-α, a known inducer of stress fiber formation in HUVECs (Figs. 5B, C, S3A). To assess whether this local order extended to coordinated alignment across the FOV, we applied circular (axial) statistics to the FibrilTool-derived orientation data. The proportion of significantly aligned FOVs (Rayleigh p < 0.05) trended higher but did not differ significantly across conditions (Friedman p = 0.0556), with 24 h TNF-α showing the greatest alignment (Fig. S3B, C). Consistently, alignment strength (R) did not vary significantly among groups (Fig. 5D).

To relate local anisotropy to global coherence, we computed Spearman correlations between per-FOV anisotropy and R. The correlation was strong under control and TNF-α conditions but weakened after 4 h EVL exposure and partially recovered at 24 h (Fig. 5E). Together, these findings indicate that while both EVL and TNF-α enhance intracellular F-actin organization, EVL transiently disrupts the coupling between local cytoskeletal order and multicellular alignment.

### Everolimus Impairs Endothelial Barrier Function as Assessed by Transendothelial Electrical Resistance and Transwell Permeability

**Fig. 6:**
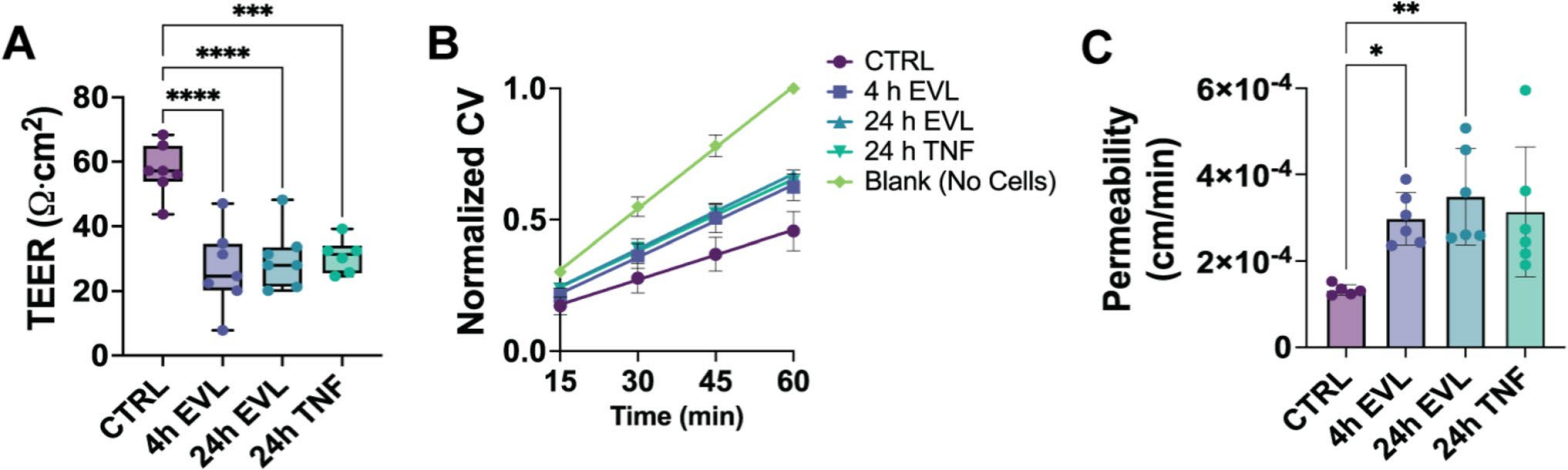
Everolimus impairs endothelial barrier function as assessed by TEER and transwell permeability. **A** Transendothelial electrical resistance (TEER) of HUVEC monolayers following treatment with vehicle control (CTRL), EVL (4 h, 24 h), or TNF-α (24 h). **B** Normalized clearance volume (CV) of FITC–dextran (4 kDa) across HUVEC monolayers at 15, 30, 45, and 60 min. Blank (no-cell) inserts served as a reference for maximal permeability. **C** Permeability coefficients calculated from clearance volume data. For TEER (**A**): N = 7 inserts per condition for CTRL, 4 h EVL, and 24 h EVL; N = 6 for TNF. For permeability (**B**, **C**): N = 5 for CTRL, and N = 6 for 4 h EVL, 24 h EVL, and TNF. Outliers in the CTRL group were identified and removed using the ROUT method (Q = 1%), as predefined in the statistical analysis plan. Data represent mean ± SD for TEER and permeability coefficients (**A**, **C**) and mean ± SEM for clearance curves (**B**), from ≥ 3 independent experiments. Statistical comparisons were performed using a Kruskal–Wallis test with Dunn’s post hoc correction. *p < 0.05; **p < 0.01; ***p < 0.001; ***p < 0.0001

To determine whether EVL-induced molecular and structural changes are associated with functional disruption of endothelial barrier integrity, we performed TEER and FITC-dextran (4 kDa) transwell permeability assays. TEER significantly decreased following both 4 h and 24 h EVL treatment, with TNF-α included as a positive control for barrier disruption (Fig. 6A). Linear regression analysis of normalized FITC-dextran clearance volume over time revealed significantly higher clearance rate constants (*k*, min⁻^1^) in EVL-treated HUVECs compared to control, indicating increased permeability (Fig. 6B). Rate constants for 4 h and 24 h EVL treatments were 0.0092 and 0.0095 min⁻^1^, respectively, versus 0.0063 min⁻^1^ in controls. All regression fits were significant (p < 0.0001), with R^2^ values exceeding 0.79. Correspondingly, permeability coefficients were 2.24- and 2.63-fold higher in 4 h and 24 h EVL-treated cells, respectively, compared to untreated controls (Fig. 6C). These functional findings support the conclusion that EVL-induced cytoskeletal and junctional remodeling contributes to measurable EBD.

## Discussion

This study builds upon prior reports that rapamycin analogs reduce VE-cadherin expression by using high-resolution confocal microscopy and quantitative image analysis to demonstrate that EVL disrupts endothelial adherens junction organization in a time-dependent manner, leading to EBD. To enhance rigor and reproducibility, we standardized imaging parameters, applied validated quantitative tools, and coupled morphological assessments with functional assays. Our findings suggest that EVL-induced junctional disruption may involve CME and lysosomal degradation, with a potential compensatory role for Golgi-dependent trafficking in restoring VE-cadherin localization at cell–cell junctions. A prominent feature of this disruption was a shift in VE-cadherin junctional conformation, prompting us to examine how these morphological changes contribute to barrier instability.

EVL treatment not only reduced VE-cadherin coverage at the cell periphery but also induced a shift in junctional conformations—most notably, a decrease in continuous and perpendicular structures and an increase in punctate structures. Punctate VE-cadherin junctions are associated with early stages of adherens junction assembly and are commonly observed during nascent contact formation [4, 37–39]. This may indicate that EVL-induced punctate junctions reflect an adaptive attempt by endothelial cells to re-establish barrier-protective contacts. However, punctate structures can also result from remodeling, in which continuous junctions fragment into discontinuous domains under stress [40]. This fragmentation-based remodeling, also observed with stimuli such as thrombin and TNF-α, suggests that EVL may trigger a comparable stress-adaptive response [40, 41].

While punctate junctions are often linked to early assembly, it remains unclear whether their increase reflects de novo formation or remodeling of existing structures. Additional markers of nascent junctions—such as VASP, fascin, or ARP2/3—were not assessed in this study [37–39, 42]. Notably, punctate junctions persisted following BFA treatment, which blocks Golgi-mediated protein delivery, suggesting that these structures can arise independently of new VE-cadherin trafficking. Moreover, analysis of early time points (1 h post-EVL) revealed reduced continuous junctions and increased punctate structures without complete junctional loss, supporting the interpretation that the 4 h phenotype primarily represents remodeling or fragmentation of existing contacts rather than de novo assembly. Together, these observations highlight that EVL-induced punctate junctions likely reflect a dynamic remodeling process driven by fragmentation of continuous contacts. The biological significance of the reduction in perpendicular conformations remains uncertain. These structures, also referred to as focal adherens junctions, may also arise from the remodeling of continuous junctions [4, 40]. In our model they comprised a small proportion of total VE-cadherin coverage, and the absolute changes were modest. Given prior associations between perpendicular junctions, vinculin recruitment, and junctional tension, future studies disrupting vinculin binding could clarify whether these shifts affect barrier integrity.

p120-catenin binds to a cluster of acidic residues in the juxtamembrane domain of VE-cadherin’s cytoplasmic tail, masking an internalization signal and thereby preventing endocytosis [43, 44]. However, rapamycin analogs have been shown to reduce p120 affinity for this domain, potentially unmasking the endocytic motif and promoting VE-cadherin internalization via clathrin-mediated pathways [18, 31]. These findings align with our observation of reduced continuous VE-cadherin at 4 h and suggest endocytic removal may underlie this early disruption. While prior work supports this hypothesis, direct evidence linking EVL to CME of VE-cadherin has been limited.

In parallel, lysosome-dependent degradation and autophagy have been implicated in the regulation of VE-cadherin. For example, knockdown of ATG7—a core autophagy regulator—preserved VE-cadherin localization following thrombin treatment [45]. Similar protective effects have been observed in models where silencing vascular endothelial protein tyrosine phosphatase (VE-PTP)—a phosphatase that stabilizes VE-cadherin and restricts leukocyte transmigration—increased endothelial permeability, but chloroquine treatment blocked VE-cadherin degradation and preserved junctional localization in this setting [32, 46, 47]. These studies suggest that internalized VE-cadherin can be targeted for lysosome-dependent degradation. Consistent with this, we found that chlorpromazine (a CME inhibitor) and chloroquine (a lysosomal inhibitor) both attenuated EVL-induced loss of continuous VE-cadherin. Together, these findings support a model in which endocytic internalization and lysosome-dependent degradation contribute to EVL-induced junctional disruption. Although supportive, these pharmacologic tools lack high specificity; future studies using genetic or orthogonal approaches will be essential to confirm the involvement of these pathways. While this study focused on CME as a primary route for VE-cadherin internalization, this does not exclude a potential role for caveolin-mediated endocytosis. Caveolin-dependent pathways have also been implicated in VE-cadherin remodeling and inflammation-induced permeability through distinct mechanisms [48]. Although not examined here, future work could explore whether caveolin-mediated trafficking counteracts or complements the EVL-induced barrier dysfunction observed in our model.

The partial recovery of VE-cadherin coverage by 24 h suggests the involvement of trafficking mechanisms. To test the role of Golgi-directed delivery, we used BFA, which inhibits anterograde transport and induces Golgi collapse [49, 50]. BFA alone reduced continuous VE-cadherin and total junctional coverage, and co-treatment with EVL and BFA led to greater disruption than EVL alone. These findings support a role for Golgi-mediated trafficking in facilitating the early reestablishment of continuous VE-cadherin at cell–cell junctions following EVL-induced disruption. This aligns with prior studies demonstrating that Golgi-derived vesicles are critical for VE-cadherin delivery and junctional maintenance [51]. Due to HUVEC sensitivity, we were unable to extend BFA exposure through the 20 h partial recovery window. As a result, we could not determine the full contribution of Golgi trafficking to VE-cadherin restoration at 24 h. We also did not assess recycling pathways (e.g., Rab11a), which have also been shown to restore surface-localized VE-cadherin [52]. Future studies examining biosynthetic and recycling pathways in parallel will be important for fully elucidating the dynamics of VE-cadherin recovery following EVL treatment.

Beyond trafficking, changes in cytoskeletal organization appear closely coupled to the morphological remodeling observed under EVL treatment. At early timepoints (e.g., 4 h), a transient rise in circularity may reflect isotropic spreading, with increased area but no significant change in perimeter. By 24 h, the correlation between increased area, perimeter, and F-actin alignment—used here as a proxy for stress fiber formation—suggests that actin remodeling may facilitate cell spreading through nuclear deformation. Prior studies have shown that stress fiber–induced compression of the nucleus reduces resistance to spreading, enabling further expansion [53–55]. While nuclear morphology was not directly assessed in this study, the observed changes in cell shape and cytoskeletal organization align with this mechanism.

Actin cytoskeletal remodeling also likely contributes to junctional reorganization. Rapamycin analogs have been shown to activate protein kinase C (PKC), which disrupts cortical actin structures and promotes VE-cadherin internalization [18, 56]. Consistent with prior findings that EVL promotes F-actin stress fiber formation through PKC- and myosin light chain kinase dependent pathways [17], we observed a time-dependent increase in F-actin anisotropy, reflecting enhanced filament alignment within individual cells. When we examined the relationship between local anisotropy and global coherence, we found a strong, predictive correlation between per-FOV anisotropy and alignment strength (R) under control and TNF-α conditions, which was completely lost after 4 h EVL treatment. This uncoupling between local cytoskeletal order and multicellular alignment likely reflects the early loss of adhesive and organizational integrity following EVL exposure. This interpretation aligns with a recent microvessel-on-chip study demonstrating that monolayer-wide actin alignment requires intact adherens junctions [57]. In that model, EDTA-mediated VE-cadherin disruption weakened coordinated actin realignment after mechanical stretch, even though individual cells retained organized stress fibers—an effect attributed to the persistence of focal adhesion–based mechanosensing independent of adherens junctions. Similarly, in our study, 4 h EVL-induced junctional disruption corresponded with the decoupling of local cytoskeletal order from multicellular actin alignment within the FOV. Consistent with the partial restoration of continuous VE-cadherin junctions at 24 h, the correlation between increasing local anisotropy and global coherence was also partially recovered.

To determine whether molecular and structural changes translated to functional outcomes, we assessed monolayer integrity using TEER and transwell permeability assays. Because TEER is susceptible to measurement variability—particularly in HUVECs, which exhibit low baseline resistance—we interpreted these results alongside FITC–dextran permeability, a complementary assay less influenced by operator bias and more directly reflective of solute flux across the monolayer. EVL significantly reduced TEER and increased 4-kDa FITC-dextran permeability, indicating that VE-cadherin disorganization, actin remodeling, and altered morphology are associated with compromised barrier function. Dysfunction was evident at both 4 and 24 h, aligning with the time course of junctional disruption and actin remodeling. These findings are consistent with prior reports showing rapamycin analogs impair endothelial barrier function [15, 17, 18]. Though TNF-α served as a classical positive control, EVL induced a greater increase in permeability, highlighting the potent barrier-disruptive effects of rapalog-based mTOR inhibition.

In addition to the limitations discussed above, several broader considerations should be acknowledged. To build upon established mechanisms linking rapalog-based mTOR inhibition to EBD, we used static culture conditions on glass to match prior studies [15, 17, 18]. However, these systems lack key mechanical cues such as shear stress and physiological matrix stiffness [58, 59]. As such, they do not fully replicate the in vivo environment. Although in vivo studies have shown rapalog-induced VE-cadherin disorganization, contextual limitations remain [10, 16, 18].

Furthermore, while our study primarily relied on high-resolution immunofluorescence imaging to assess VE-cadherin organization, we took care to minimize common methodological limitations by using standardized acquisition settings, validated morphometric tools (e.g., JAnaP), and complementary functional assays (TEER and transwell permeability). These measures helped ensure objective and reproducible quantification of junctional organization and barrier function. Beyond these technical considerations, observations in HBMECs revealed a more sustained EVL response compared to HUVECs, which recovered by 24 h. This suggests vascular bed–specific differences that merit further study.

Recent advancements in mTOR inhibitors, such as Torin-2, have shown promise in mitigating EBD in preclinical models [16, 18]. Unlike EVL, Torin-2 directly inhibits both mTORC1 and mTORC2 [60, 61], but the specific impact of mTORC2 inhibition on adherens junction integrity and barrier function remains unclear. Future studies employing selective mTORC1 versus mTORC2 inhibitors, alongside genetic manipulation of trafficking machinery, will be essential to delineate the full spectrum of mTOR pathway effects and clarify the role of Golgi trafficking in VE-cadherin restoration. Additionally, ex vivo analysis of VE-cadherin conformations and validation in relevant animal models will be critical for enhancing the translational relevance of these findings.

In conclusion, our findings demonstrate that EVL disrupts endothelial junction organization by promoting VE-cadherin internalization, potentially via CME and lysosome-dependent degradation. This disruption is accompanied by cytoskeletal and morphological changes, leading to measurable EBD. We further show that Golgi-mediated trafficking may help restore adherens junctions. By linking molecular, structural, and functional changes, this study provides mechanistic insight into how EVL compromises vascular integrity and highlights the need to explore these effects across vascular beds and translational models.

## Supplementary Information

Below is the link to the electronic supplementary material.

Supplementary file1 (DOCX 1635 kb)

## Data Availability

All raw data are available upon request.
