# Supplementary material for: Endocytic Pathways and Actin Remodeling Mediate Everolimus-Induced VE-Cadherin Disorganization and Barrier Dysfunction
Source: Cell Mol Bioeng. 2025 Dec 3;19(1):43–59. doi: 10.1007/s12195-025-00881-y (PMC12915895; doi:10.1007/s12195-025-00881-y)
Supplement: Supplementary file 1 — Supplementary file1 (DOCX 1635 kb) [file 12195_2025_881_MOESM1_ESM.docx]

**Endocytic Pathways and Actin Remodeling Mediate Everolimus-Induced VE-Cadherin Disorganization and Barrier Dysfunction**

Ken D. Brandon^1^, Yoshi Chettri^1^, Azkah Anjum^1^, and Kimberly M. Stroka^1,2,3,4#^

1) Fischell Department of Bioengineering, University of Maryland, College Park, MD 20742, USA

2) Marlene and Stewart Greenebaum Comprehensive Cancer Center, University of Maryland – Baltimore, MD 21201, USA

3) Biophysics Program, University of Maryland, College Park, MD 20742, USA

4) Center for Stem Cell Biology and Regenerative Medicine, University of Maryland – Baltimore, MD, 21201, USA

# Corresponding Author:

Kimberly M. Stroka

3110 A. James Clark Hall

Fischell Department of Bioengineering

University of Maryland, College Park

8278 Paint Branch Drive

College Park, MD 20742

TEL: 301-314-1813

FAX: 301-405-9953

EMAIL: [kstroka@umd.edu](mailto:kstroka@umd.edu)

### **Orchid ID**

Ken: <https://orcid.org/0009-0004-2244-2747>

Yoshi: <https://orcid.org/0000-0003-1215-7673>

Azkah: n/a

Kimberly: <https://orcid.org/0000-0003-3314-2067>

### **Acknowledgments**

This work was supported by a National Science Foundation CAREER Award (No. 1944121 to K.M.S.), NIGMS MIRA #R35GM142838 (to KMS), a Clark Doctoral Fellowship (to K.D.B.), and the National Institutes of Health, National Heart, Lung, and Blood Institute (F31HL178291 to K.D.B.).

**Table S1. Pairwise statistical comparisons supporting VE-cadherin preservation by endocytic and lysosomal pathway inhibition.**

Adjusted p-values are shown for all treatment group comparisons at 0, 1, 2, and 4 hours after everolimus (EVL) treatment. Data correspond to the quantifications in Figure 2C–F. Treatment groups include EVL-only (E), chlorpromazine + EVL (C+E), chloroquine + EVL (Q+E), and chlorpromazine + chloroquine + EVL (C+Q+E). Statistical analysis was performed using the Kruskal–Wallis test with Dunn’s post hoc correction. Statistically significant differences are indicated in teal.

|  | **Adjusted P value** | | | | |
| --- | --- | --- | --- | --- | --- |
| **h** |  | **Continuous Junction** | **Punctate Junction** | **Perpendicular Junction** | **Monolayer Coverage** |
| **0** | E vs. C+E | >0.9999 | >0.9999 | >0.9999 | >0.9999 |
|  | E vs. Q+E | >0.9999 | >0.9999 | >0.9999 | >0.9999 |
|  | E vs. C+Q+E | >0.9999 | >0.9999 | >0.9999 | >0.9999 |
|  | C+E vs. Q+E | >0.9999 | >0.9999 | >0.9999 | >0.9999 |
|  | C+E vs. C+Q+E | >0.9999 | >0.9999 | >0.9999 | >0.9999 |
|  | Q+E vs. C+Q+E | >0.9999 | >0.9999 | >0.9999 | >0.9999 |
| **1** | E vs. C+E | <0.0001 | <0.0001 | <0.0001 | <0.0001 |
|  | E vs. Q+E | <0.0001 | 0.0015 | >0.9999 | <0.0001 |
|  | E vs. C+Q+E | <0.0001 | <0.0001 | >0.9999 | <0.0001 |
|  | C+E vs. Q+E | >0.9999 | 0.001 | <0.0001 | 0.6374 |
|  | C+E vs. C+Q+E | >0.9999 | 0.0366 | 0.0082 | 0.0042 |
|  | Q+E vs. C+Q+E | >0.9999 | >0.9999 | 0.4512 | 0.4143 |
| **2** | E vs. C+E | <0.0001 | 0.0013 | >0.9999 | <0.0001 |
|  | E vs. Q+E | <0.0001 | 0.0027 | 0.0008 | >0.9999 |
|  | E vs. C+Q+E | <0.0001 | <0.0001 | 0.0293 | 0.0075 |
|  | C+E vs. Q+E | >0.9999 | >0.9999 | <0.0001 | 0.0006 |
|  | C+E vs. C+Q+E | 0.1058 | >0.9999 | 0.1962 | 0.3878 |
|  | Q+E vs. C+Q+E | 0.0254 | >0.9999 | <0.0001 | 0.1166 |
| **4** | E vs. C+E | <0.0001 | <0.0001 | 0.0144 | <0.0001 |
|  | E vs. Q+E | <0.0001 | <0.0001 | >0.9999 | <0.0001 |
|  | E vs. C+Q+E | <0.0001 | <0.0001 | <0.0001 | <0.0001 |
|  | C+E vs. Q+E | >0.9999 | >0.9999 | 0.0022 | 0.1356 |
|  | C+E vs. C+Q+E | 0.9724 | 0.1822 | 0.0283 | >0.9999 |
|  | Q+E vs. C+Q+E | 0.9431 | >0.9999 | <0.0001 | 0.0044 |

###

### **FIGURES**

**
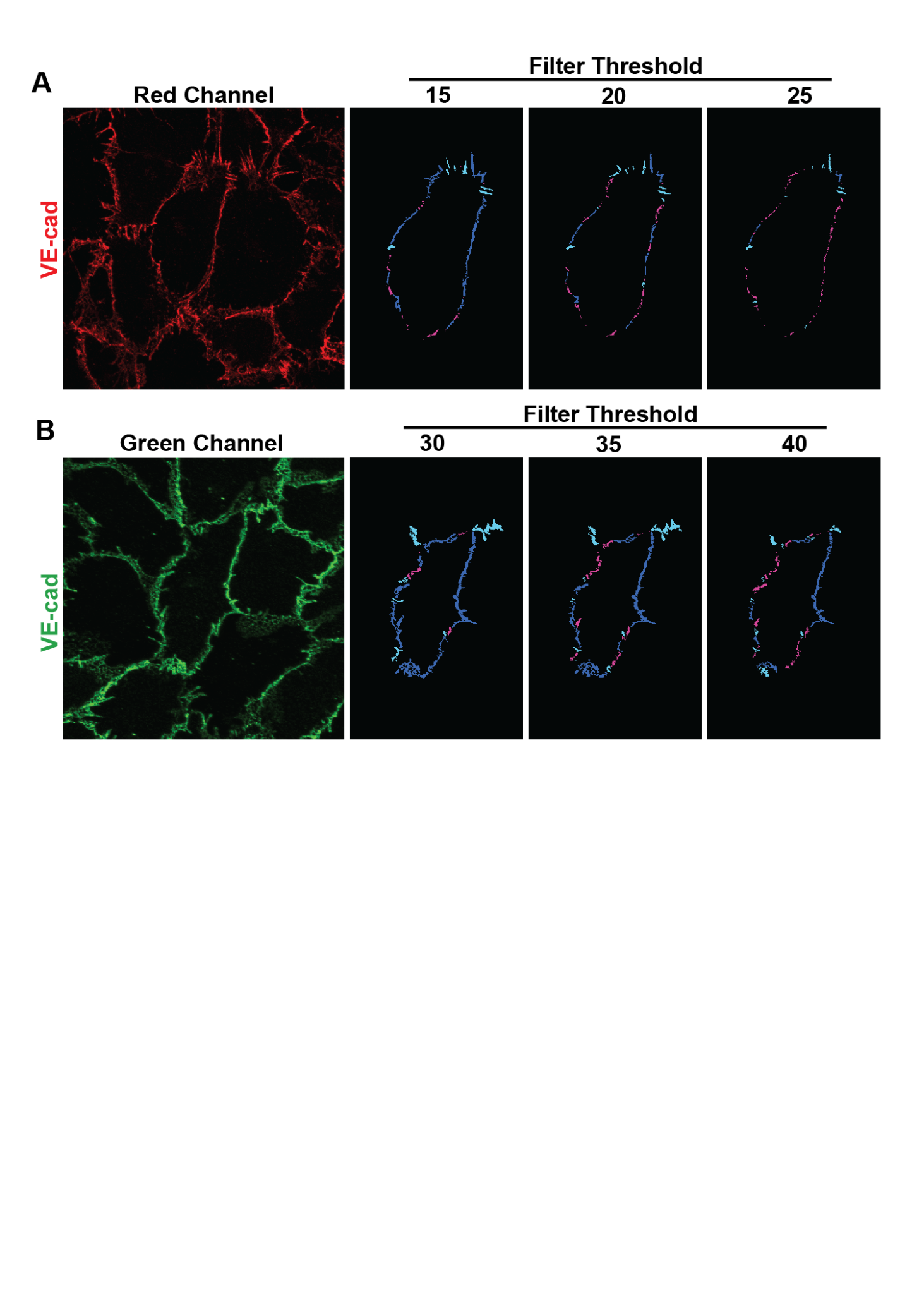
**

**Figure S1.** Jupyter notebook thresholding workflow. (A) Thresholding output for VE-cadherin-labeled HUVECs imaged in the red channel. Left: representative raw image with boxed region indicating area analyzed. Right: comparison of JAnaP classification output at three filter thresholds (15, 20, and 25). A threshold of 20 was selected based on optimal segmentation fidelity and limited background noise. (B) Thresholding output for VE-cadherin-labeled HUVECs imaged in the green channel. Left: representative raw image with boxed region indicating area analyzed. Right: JAnaP classification output across thresholds (30, 35, and 40). A filter threshold of 35 was selected for green-channel datasets for the same reason. These thresholds were standardized for all subsequent analyses.


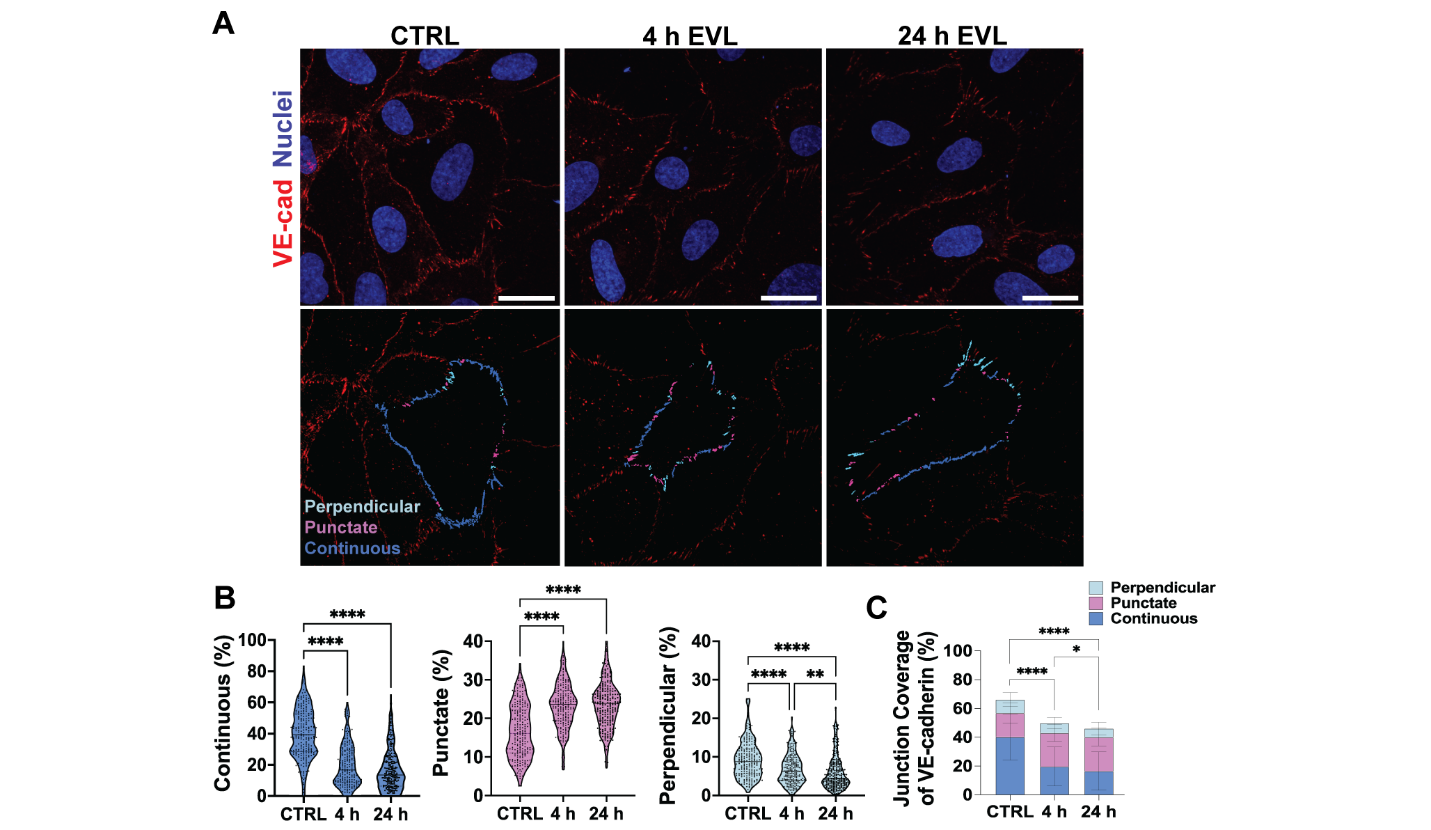


**Figure S2.** VE-cadherin conformations in HBMECs following Everolimus treatment. (A) Representative images showing VE-cadherin (VE-cad, red) and nuclei (blue) in HBMECs under control (CTRL), 4 h EVL, and 24 h EVL treatment conditions, with JAnaP segmentation overlayed, color-coded by junction classification: continuous (blue), punctate (pink), and perpendicular (cyan). (B) Quantification of VE-cadherin conformations—continuous, punctate, and perpendicular—using the JAnaP (n = 217–236 cells per condition) (C) Population-averaged junction coverage of VE-cadherin, shown as the mean proportions of continuous, punctate, and perpendicular conformations across the sample cell population. All data represent mean ± SD from three independent experiments. A Kruskal–Wallis test with Dunn’s post hoc was used for analysis. Scale bars: 25 µm. Images acquired at 60X and cropped; calibration preserved. *p < 0.05; **p < 0.01; ***p < 0.001; ****p < 0.0001.


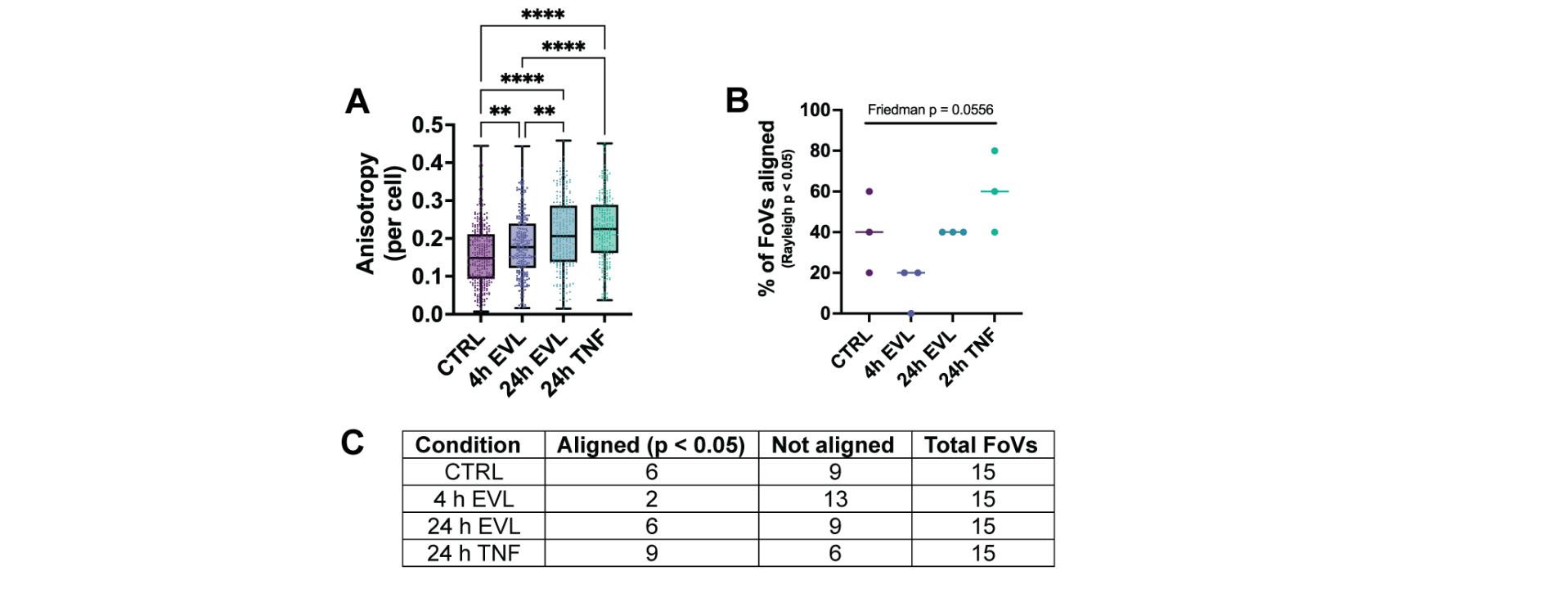


**Figure S3.** Quantification of F-actin anisotropy and proportion of aligned fields of view following Everolimus treatment. (A) Per-cell F-actin anisotropy quantified using FibrilTool from F-actin–immunostained HUVECs. Higher anisotropy values indicate greater local alignment of actin fibers within individual cells. (B) Percentage of fields of view (FOVs) exhibiting significant directional alignment, determined by the Rayleigh test (p < 0.05). Across three independent experiments (n = 15 FOVs per condition), the proportion of aligned FOVs showed a non-significant trend toward increase with 24 h TNF-α treatment (Friedman p = 0.0556; Dunn’s post hoc p > 0.05 for all pairwise comparisons). (C) Summary table showing the number of FOVs classified as aligned or not aligned based on the Rayleigh test. Statistical analyses were performed using Kruskal–Wallis test (A) and Friedman test with Dunn’s post hoc correction (B–C). *p < 0.05; **p < 0.01; ***p < 0.001; ****p < 0.0001.
